# Development of a fumonisin-sensitive *Saccharomyces cerevisiae* indicator strain and utilization for activity testing of candidate detoxification genes

**DOI:** 10.1128/aem.01211-23

**Published:** 2023-12-06

**Authors:** Tamara Krska, Krisztian Twaruschek, Nina Valente, Rudolf Mitterbauer, Dieter Moll, Gerlinde Wiesenberger, Franz Berthiller, Gerhard Adam

**Affiliations:** 1 Department of Applied Genetics and Cell Biology, Institute of Microbial Genetics, University of Natural Resources and Life Sciences, Vienna (BOKU), Tulln, Austria; 2 Austrian Competence Centre for Feed and Food Quality, Safety & Innovation, FFoQSI GmbH, Tulln, Austria; 3 dsm-firmenich ANH Research Center Tulln, Tulln, Austria; 4 Department of Agrobiotechnology, Institute of Bioanalytics and Agro-Metabolomics, IFA-Tulln, University of Natural Resources and Life Sciences, Vienna (BOKU), Tulln, Austria; Washington University, St. Louis, Missouri, USA

**Keywords:** bioassay, mycotoxin detoxification, bioindicator, *S. cerevisiae*

## Abstract

Fumonisins, produced mainly by various *Fusarium* species, are sphinganine-analog mycotoxins and potent inhibitors of ceramide synthases in animals, plants, and other eukaryotes, leading to toxicity and programmed cell death. Wild-type baker’s yeast is naturally highly resistant to fumonisin B_1_ (FB1); on rich medium, it tolerates more than 1,000 µM. We report the construction of a fumonisin-sensitive *Saccharomyces cerevisiae* strain with six disrupted genes: *SNQ2*, *PDR12*, and *YOR1*, coding for ATP-binding cassette transporters involved in active efflux; *CKA2*, encoding casein kinase II, regulating ceramide synthase activity by phosphorylation; and *LCB3* and *VPS51*, encoding proteins involved in the biosynthesis and recycling of sphingolipid precursors, respectively. The constructed FB1-sensitive strain YTKT33 was tested on complex yeast extract-peptone-dextrose (YPD) and synthetic complete medium alongside the wild-type strain and strains with only three (YRU74) or five (YRU94ML) disrupted genes. YTKT33, exhibiting full growth inhibition at a concentration below 5 µM FB1 on synthetic complete agar lacking uracil (SC-URA), was used as a host for heterologous expression of previously reported fumonisin detoxification genes. Transformants expressing either the *Sphingopyxis fumD* esterase, which removes removing tricarballylic acid side chains, or an *Aspergillus* amine oxidase, which converts the amino- to a keto-group, can grow at FB1 concentrations inhibitory for transformants containing the empty vector. YTKT33 was inhibited by other type B fumonisins (B_2_, B_3_, B_4_) at concentrations very similar to FB1, allowing rapid evaluation of total fumonisin toxicity in crude fungal extracts.

**IMPORTANCE:**

Fumonisins can cause diseases in animals and humans consuming *Fusarium*-contaminated food or feed. The search for microbes capable of fumonisin degradation, or for enzymes that can detoxify fumonisins, currently relies primarily on chemical detection methods. Our constructed fumonisin B1-sensitive yeast strain can be used to phenotypically detect detoxification activity and should be useful in screening for novel fumonisin resistance genes and to elucidate fumonisin metabolism and resistance mechanisms in fungi and plants, and thereby, in the long term, help to mitigate the threat of fumonisins in feed and food.

## INTRODUCTION

Fumonisins are a class of toxicologically relevant *Fusarium* secondary metabolites that are produced predominantly by members of the *Fusarium fujikuroi* species complex (1). Particularly important are *Fusarium verticillioides*, *Fusarium proliferatum*, and other species (*Fusarium temperatum*, *Fusarium subglutinans*) on maize in warmer climates, such as in Africa (2), where fumonisin was discovered. Fumonisin production and contamination also occur in southern parts of Europe, not only on maize but also in wheat and barley (3–5). Fumonisin-producing *Fusarium proliferatum* can also infect rice (6), where fumonisin production is a virulence factor in rice spikelet rot disease (7). While fumonisin production contributes to virulence in maize seedlings (8), it is dispensable for the ability to cause maize ear rot (9). Climate change seems to increase the previously rather negligible threat of fumonisin contamination in central Europe (10).

Fumonisins are sphinganine-analog mycotoxins with a sphinganine-like backbone structure (11). They target ceramide synthases (very-long-chain fatty acyl-CoA:sphingoid base *N*-acyltransferases) of animals (12), plants (13) and fungi and can trigger programmed cell death in animal cells and plant cells (14–16).

To characterize the toxicity of fumonisins and to develop mitigation strategies, mostly chemical assay methods, requiring expensive equipment, are used since previously established bioassays for fumonisin toxicity have shortfalls. Highly purified toxin preparations are needed for mammalian cells cultured *in vitro* to avoid the unspecific toxic effects of other metabolites co-occurring in crude toxin extracts. The reported susceptibility of different human and animal cell lines is quite variable. The effective concentrations ranged mainly from 10 to 100 µM (17). The IC_50_ value for primary rat hepatocytes was reported to be 2,000 µM (18), while human HepG2 cells are more sensitive, with an IC_50_ value of 399 µM (19). Plants are typically very sensitive; e.g., *Arabidopsis* seed germination and root development are already strongly inhibited by 1 µM fumonisin B_1_ (FB1) (20). However, such bioassays are rather time-consuming (e.g., requiring a 10-day observation period). Duckweed (*Lemna minor*) growth inhibition by 40 µM of fumonisin metabolites over a 5-day period was used to investigate the structure-activity relationship of different fumonisins and metabolites (21). Similarly, labor-intensive and low-throughput whole-animal test systems were described. For instance, a 4-day incubation period was needed to observe the death or disintegration of *Hydra attenuata* during exposure to 150 µg/mL (~208 µM) FB1 (22). Similarly, microscopic observation of non-motile or dead brine shrimp (*Artemia salina*) yielded IC_50_ values for FB1 ranging from 1.7 µM (48 h) to about 10 µM after 24 h (23, 24).

Adding detoxification enzymes to fumonisin-contaminated feed commodities (25) or expressing heterologous detoxification genes already in transgenic plants (26) are possible strategies to counteract and reduce mycotoxin contamination. However, testing enzyme activity *in vitro* is a major bottleneck in the development of such agents. As of now, *in vitro* efficacy is verified through the determination of the detoxified products [e.g., hydrolyzed FB1 (HFB1)], which requires costly and laborious liquid chromatography-mass spectrometry (LC-MS). Since little is known about fumonisin metabolism in plants, useful endogenous detoxification genes may exist that could be selected by breeders or manipulated by gene editing—the search is ongoing. To the best of our knowledge, none of the previously mentioned test organisms can serve as convenient hosts for the expression and high-throughput screening of potential candidate detoxification genes. We therefore set out to engineer *Saccharomyces cerevisiae* as an indicator organism for monitoring the toxicity of fumonisins and to phenotypically detect and test expressed detoxification enzymes.

## RESULTS

### Strain development with a crude extract containing fumonisins

**FIG 1:**
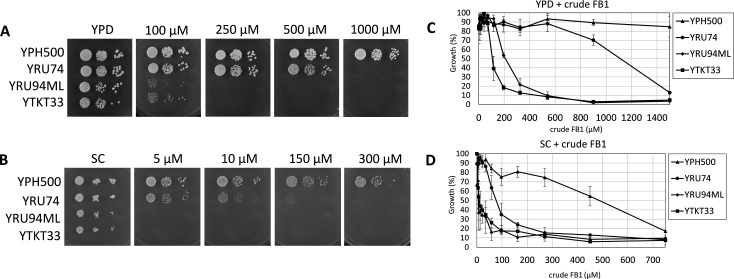
Growth inhibition of yeast by a crude fumonisin-containing extract (“crude FB1”) on solid and in liquid media. The wild-type yeast laboratory strain YPH500 and three derived multi-deletion strains YRU74 (triple ABC transporter mutant, *yor1 pdr12 snq2*), YRU94ML (*yor1 pdr12 snq2* plus *cka2* and *lcb3*), and YTKT33 (*yor1 pdr12 snq2 cka2 lcb3* plus *vps51*) were grown in complex (YPD) and synthetic (SC) media overnight and rediluted to an OD_600_ of 0.1 in the morning. After reaching (OD_600_ >0.3), they were diluted again for spotting on (**A**) YPD and (**B**) SC plates containing crude FB1, as indicated. (**C** and **D**) The inoculum was pipetted into a 96-well microtiter plate with different concentrations of crude FB1 and put into an incubator, where the absorbance at OD_600_ was measured after 24 h. At least three replicates were used for each strain. On the x-axis, the graph shows the final FB1 concentration that the strains were exposed to, while the y-axis shows the inhibition of growth in % compared to each strain growing without exposure to FB1. The standard deviation was calculated for the replicates and displayed in the error bars.

Wild-type *Saccharomyces cerevisiae* is highly suited as a host for heterologous gene expression and for monitoring the toxicity of various substances on agar media or in liquid culture on microtiter plates. Yet, *S. cerevisiae* is highly resistant to many compounds, including FB1. To save costly pure FB1 during our initial characterization and strain improvement, we used a crude extract containing fumonisins, which was prepared from maize cultures inoculated with various *Fusarium* strains (see Materials and Methods), with an FB1 content of 3,180 mg/L as determined by LC-MS/MS. Based on this concentration value, the “wild-type” laboratory strain YPH500 (see Table 1 for relevant genotype and reference) tolerated more than 1,000 µM on rich medium (yeast extract-peptone-dextrose, YPD) (Fig. 1A and C) and more than 300 µM on synthetic complete (SC) medium (Fig. 1B and D). As it has been reported that the ATP-binding cassette (ABC) transporter *YOR1* (Yeast Oligomycin Resistance) is involved in fumonisin resistance (27), we generated and tested strains that combined *yor1* with mutations of other ABC transporters that were implicated in mediating resistance to charged compounds (28). We generated all double mutants and the triple mutant with the genes *SNQ2* (Sensitivity to 4-NitroQuinoline-N-oxide) and *PDR12* (Pleiotropic Drug Resistance), and continued strain improvement with the triple mutant YRU74 (see Table 1 for strain genotypes). This strain had reduced FB1 resistance both on YPD and SC medium compared to wild-type on solid agar plates (Fig. 1A and B). We also tested growth in liquid media in microtiter plates, and here (Fig. 1C and D) resistance was lower.

**TABLE 1:** Yeast strains used or generated in this study

Name	Derived from/alteration: plasmid (primers)	Genotype
YPH499	Wild-type lab strain	*MATa ura3-52 lys2-801 ade2-101 trp1-Δ63 his3-*Δ*200 leu2-*Δ*1*
YPH500	Wild-type lab strain	*MATα ura3-52 lys2-801 ade2-101 trp1-*Δ*63 his3-*Δ*200 leu2-*Δ*1*
YYM5	YPH500/pYM25, FOA	*MATα snq2::hisG*
YRE106	YPH499/pYM63	*MATa pdr12::hisG-URA3-hisG*
YRU75	YRE106 FOA	*MATa pdr12::hisG*
YZGA456	YPH499 pDK30	*MATa yor1::hisG-URA3-hisG*
YZGA494	YPH499 pDK30, FOA	*MATa yor1::hisG*
YRE108	YMM5/pYM63	*MATα snq2::hisG pdr12::hisG-URA3-hisG*
YZGA538	YRE108, FOA	*MATα snq2::hisG pdr12::hisG*
YTKT34	YYM5 pDK30	*MATα snq2::hisG yor1::hisG-URA3-hisG*
YTKT35	YRU75/pDK30	*MATa pdr12:hisG yor1::hisG-URA3-hisG*
YTKT60	YTKT34, FOA	*MATα snq2::hisG yor1::hisG*
YTKT61	YTKT35, FOA	*MATα pdr12::hisG yor1::hisG*
YZGA1208	YZGA538/pDK30	MATα *snq2::hisG pdr12::hisG yor1::hisG-URA3-hisG*
YRU74	YZGA1208, FOA	MATα *snq2::hisG pdr12::hisG yor1::hisG*
YRU81	YRU74/pUG72 (dcka2-FW + dcka2 RV)	MATα *snq2::hisG pdr12::hisG yor1::hisG cka2*Δ*::loxP-KlURA3-loxP*
YRU83	YRU74/pUG72 (dlcb3-FW + dlcb3 RV)	MAT*α snq2::hisG pdr12::hisG yor1::hisG lcb3*Δ*::loxP-KlURA3-loxP*
YRU93	YRU83/pUG73 (dcka2-FW + dcka2 RV)	*MATα snq2::hisG pdr12::hisG yor1::hisG lcb3*Δ*::loxP-KlURA3-loxP cka2*Δ*::loxP-KlLEU2-loxP*
YRU94	YRU81/pUG73 (dlcb3-FW + dlcb3 RV)	*MATα snq2::hisG pdr12::hisG yor1::hisG cka2*Δ*::loxP-KlURA3-loxP lcb3*Δ*::loxP-KlLEU2-loxP*
YRU94*	YRU94 from YPG, red on YPD	*MATα snq2::hisG pdr12::hisG yor1::hisG cka2*Δ*::loxP-KlURA3-loxP lcb3*Δ*::loxP-KlLEU2-loxP*
YRU94ML-A	YRU94* pOS4a ADE+/YPGAL	*MATα snq2::hisG pdr12::hisG yor1::hisG cka2*Δ::*loxP lcb3*Δ*::loxP* [pOS4a: *ADE2-P_GAL1_-Cre*]
YRU94ML	YRU94ML ade2	*MATα snq2::hisG pdr12::hisG yor1::hisG cka2*Δ*::loxP lcb3*Δ*::loxP*
YTKT1	YRU94ML, YPGal	*MATα snq2::hisG pdr12::hisG yor1::hisG cka2*Δ*::loxP lcb3*Δ*::loxP vps51*Δ*::loxP-KlLEU2-loxP*
YTKT33	YTKT1 pBS49, YPGal, plasmid loss	*MATα snq2::hisG pdr12::hisG yor1::hisG cka2*Δ*::loxP lcb3*Δ*::loxP vps51*Δ*::loxP*

Since the resistance level of YRU74 was still unacceptably high, requiring concentrations of FB1 exceeding naturally occurring levels, we next inactivated the *CKA2* gene, encoding one of the catalytic subunits (alpha′) of casein kinase 2. It has been reported that such a mutant had 3–4× reduced ceramide synthase activity and reduced resistance to FB1 (29). We have furthermore knocked out *LCB3*, encoding one of the two long-chain base-1-phosphate phosphatases (paralog *YSR3*), which are needed for the utilization of exogenous long-chain base phosphates present in rich medium. The *cka2 lcb3* double mutant YRU94 initially segregated small and large colonies upon revival from −80°C stocks, so a stable respiration-competent clone was selected on YPG, which also formed stable red colonies on YPD, indicating an unimpaired respiration needed for accumulation of the red pigment in adenine-requiring *ade2* mutants. Transformation markers (*LEU2* and *URA3*) were removed from this strain, designated as YRU94*, using plasmid pOS4a (*ADE2 GAL1-Cre*), resulting in YRU94ML-A (ura^−^ and leu^−^ but still ADE^+^). After the loss of the pOS4a plasmid, the strain YRU94ML was obtained, which clearly exhibited an over fivefold increase in sensitivity toward FB1 as compared to the *yor1 snq2 pdr12* parental strain (Fig. 1C).

**FIG 2:**
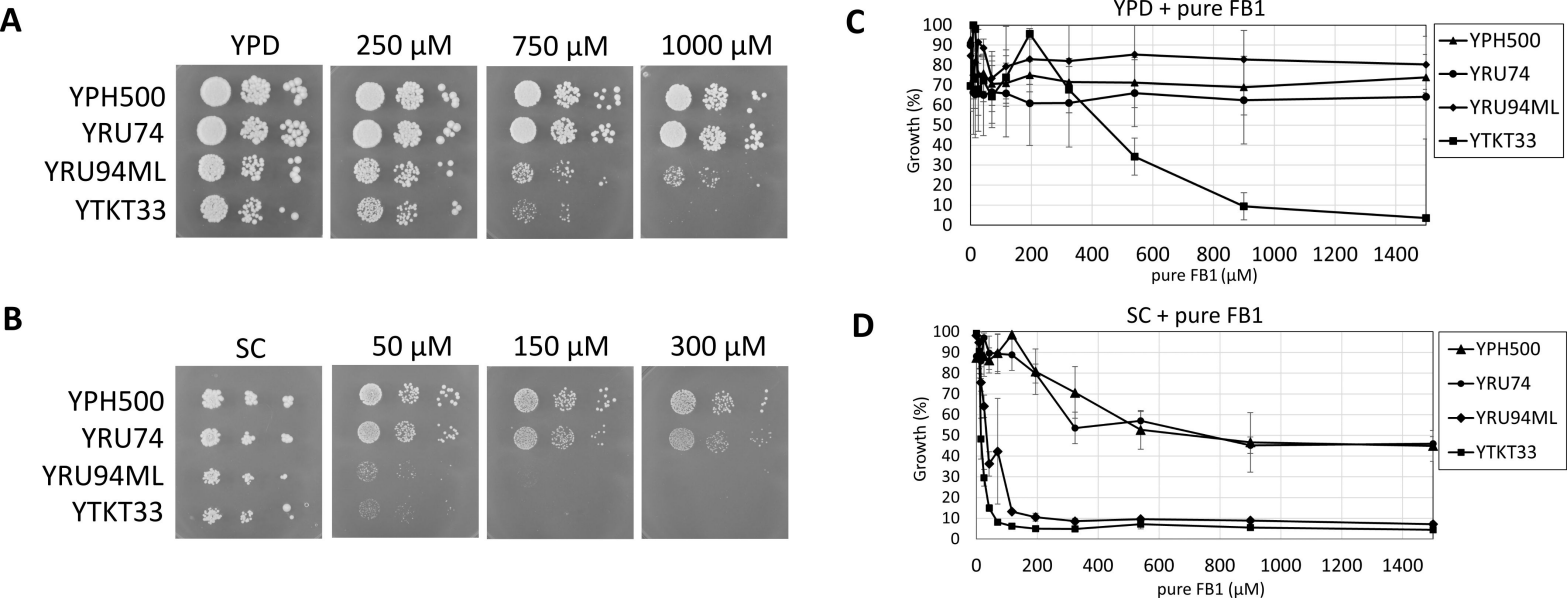
Sensitivity testing with pure FB1. For strain genotypes, refer to Fig. 1 or Table 1. Growth inhibition of yeast by a 98% pure FB1 stock on solid and liquid media. The wild-type yeast laboratory strain YPH500 and three derived multi-deletion strains YRU74 (triple ABC transporter mutant, *yor1 pdr12 snq2*), YRU94ML (*yor1 pdr12 snq2* plus *cka2* and *lcb3*), and YTKT33 (*yor1 pdr12 snq2 cka2 lcb3* plus *vps51*) were grown in complex (YPD) and synthetic (SC) media overnight and rediluted to an OD_600_ of 0.1 in the morning. After reaching OD_600_ >0.3, they were diluted again for spotting on (**A**) YPD and (**B**) SC plates containing crude FB1, as indicated. (**C and D**) Inoculum was pipetted into a 96-well microtiter plate with different concentrations of pure FB1 and put into an incubator, where the absorbance at OD_600_ was measured after 24 h. At least three replicates were used for each strain.

To further reduce FB1 resistance, we also disrupted *VPS51* (Vacuolar Protein Sorting), a gene encoding a component of the Golgi-associated retrograde protein complex. Endosome-to-Golgi retrograde vesicular transport allows recycling of complex sphingolipids (30), which would otherwise be degraded using the default pathway in the vacuole. The reduced sensitivity of the strain with the additional *vps51* mutation to FB1 is evident from Fig. 1C (strain grown in liquid YPD) and in the spotting experiment with purified FB1 (Fig. 2), where strain YTKT33 (*yor1 snq2 pdr12 cka2 lcb3 vps51*) is more sensitive than its precursor YRU94ML with wild-type *VPS51* both in YPD liquid culture (Fig. 2C) and on YPD plates (Fig. 2A).

### Testing the constructed strains with purified FB1

The constructed strains were subsequently exposed to media spiked with purified, commercially available FB1 (>98% purity, Fermentek). Compared to Fig. 1, in which only crude fumonisin extracts were used, it is evident in Fig. 2 that higher levels of the pure toxin are needed to achieve a similar degree of inhibition. One obvious explanation is that the crude extract contains other intermediates of FB1 synthesis, such as fumonisins B_2_ (FB2), B_3_ (FB3), and B_4_ (FB4), and possibly additionally other forms, such as non-toxic forms (e.g., *N*-acetylated fumonisin A), that might be hydrolyzed and reactivated by the yeast. Yet, no fumonisin A metabolites were detected by LC-MS/MS in the crude extract, and later results (see below) indicate that the response to FB2, FB3, and FB4 was similar to FB1 (see Fig. 4).

The different behavior of the mutants in the two media is also of interest. While on SC medium, YRU94ML behaved similar to YTKT33 (Fig. 2B and D), on the rich medium, the latter strain was far more susceptible, indicating that components (sphingolipid metabolites not present in SC medium but presumably in the yeast extract in YPD) could reduce fumonisin toxicity in a *VPS51*-dependent manner.

### Dose-response of strain YTKT33 to B-type fumonisins in liquid synthetic medium

**FIG 3:**
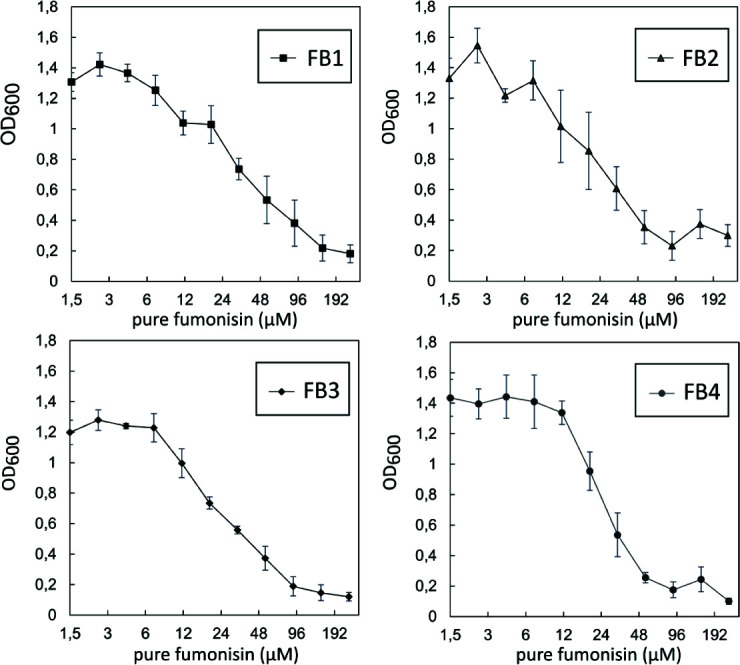
Growth inhibition of YTKT33 in microtiter plates in SC medium by B-type fumonisins FB1-FB4. The sensitive yeast strain YTKT33 was grown in SC medium and exposed to different concentrations of FB1, FB2, FB3, and FB4. Strain inoculum was pipetted into a microtiter well plate with 0.6^N^ dilutions of the respective fumonisins. After 24 h at 30°C, the optical density at 600 nm (OD_600_) was measured to monitor growth. The blank (medium without yeast) was subtracted from the measured OD_600_ values. Means and standard deviations were calculated for four replicates each, as depicted in the error bars of the graphs.

To determine the inhibition characteristics of different B-type fumonisins [which differ in the hydroxylation pattern; for review, see reference (31)], we obtained high-purity FB1, FB2, FB3, and FB4 and tested their potency to inhibit the growth of yeast strain YTKT33 in liquid SC medium in microtiter plates. The inhibition of strain YTKT33 upon exposure to fumonisins B_2_, B_3_, and B_4_ was similar to the inhibition by FB1 (see Fig. 3).

**FIG 4:**
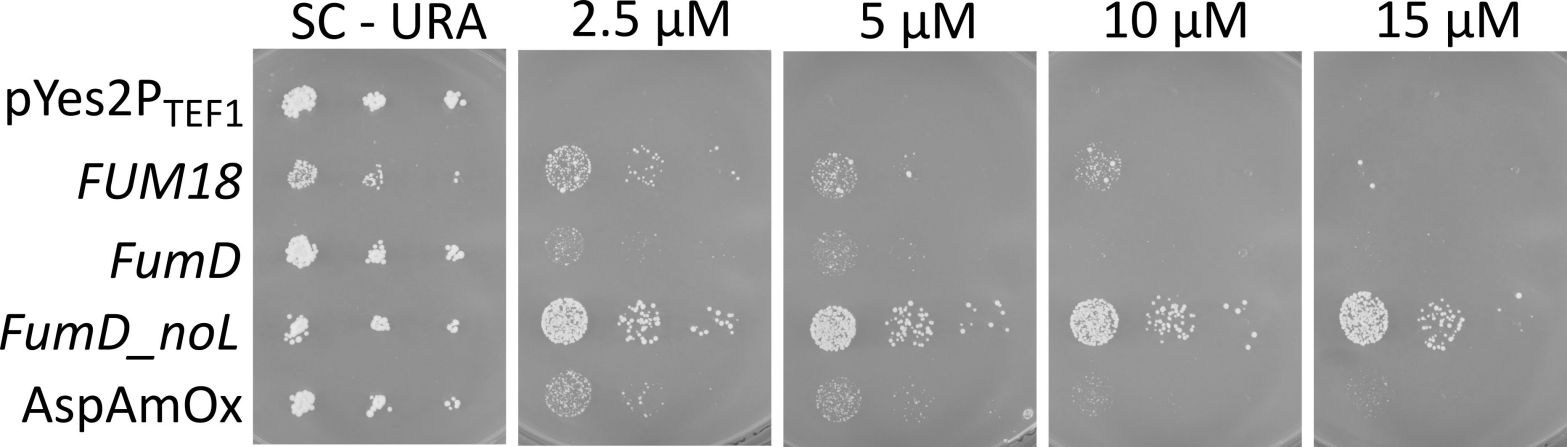
Growth of YTKT33-derived strains on URA-dropout SC agar media containing increasing concentrations of FB1 carrying either the empty expression vector pYes2-P_TEF1_ (negative control) or expressing one of the following genes: *FUM18* (ceramide synthase from the *F. verticillioides* fumonisin cluster, positive control), *FumD* (*Sphingopyxis* fumonisin carboxylesterase *FumD*), *FumD_noL* (*FumD* lacking the N-terminal secretion signal), and AspAmOx (*Aspergillus* amine oxidase). Three dilutions of the transformed YTKT33 strain were spotted for each culture (OD_600_ = 0.1, 0.01, and 0.001).

As shown in Fig. 3, the IC_50_ values for the four tested fumonisins, FB1, FB2, FB3, and FB4, were all observed to be between 24 and 26 µM. The relative composition of the different compounds in the extract can be variable depending on the *Fusarium* strain and harvesting time, but due to the similar dose-response, the toxicity reflects the sum of all B-type fumonisins. The inhibitory concentrations for YTKT33 obtained in liquid SC culture (in microtiter plates with limited aeration in still cultures) were clearly higher than the inhibitory concentrations observed for the empty vector transformant in spottings carried out on solid synthetic complete media lacking uracil (SC-URA, see Fig. 4).

### Heterologous expression of fumonisin detoxification genes

Strain YTKT33 is growing slower than the YPH500 precursor strain. Alterations in sphingolipids have been reported to negatively affect heat shock resistance (32). However, YTKT33 is robust and easily transformable with the standard lithium transformation protocol, which includes a heat shock treatment. We utilized the strain as a host to express candidate fumonisin resistance genes. The empty vector pYes2-P_TEF1_ was used in a previous study to express candidate ceramide synthases from a fumonisin-producing *F. verticillioides* strains behind the constitutive *TEF1* promoter (33). In the aforementioned study, evidence was provided that the *FvFUM18* gene, located in the fumonisin biosynthetic cluster, codes for an insensitive ceramide synthase providing fumonisin self-resistance (33). The empty vector and the *FUM18* expression vector were used as negative and positive controls in our study. As evident from Fig. 4, the lowest tested fully inhibitory concentration is clearly below 5 µM FB1, at approx. 2.5 µM FB1, which is about 10-fold lower than the IC_50_ in liquid SC medium in microtiter plates. Plasmid-based expression of *FUM18* in YTKT33 results in an approximately fourfold increase in resistance, up to 10 µM FB1.

In addition, we expressed two enzymes altering the chemical structures of fumonisin B_1_, leading to its detoxification. First, we expressed the *Sphingopyxis macrogoltabida* MTA144 *fumD* gene (34), which encodes a periplasmic secreted carboxylesterase that removes the tricarballylic acid side chains of fumonisins and leads to the less toxic metabolite HFB1 (34). We have constructed two different *fumD* expression vectors, with and without the N-terminal secretion signal. While complete *fumD* expression also leads to a low increase in resistance phenotype, the strain lacking the signal peptide (fumD_noL) exhibits high-level FB1 resistance (see Fig. 4). Previously, another detoxification gene from *Aspergillus*, encoding a flavine-dependent amine oxidase, had been described (35, 36). This enzyme converts the amino group of fumonisins into a keto group. We have custom-synthesized the amine oxidase gene with an identical sequence as previously reported (35) and cloned it behind the *TEF1* promoter. The expression of this gene (without the 6xHis epitope tag in Fig. 4) also led to increased fumonisin B_1_ resistance, although at a lower level than YTKT33 expressing *fumD_noL*. In summary, these results show that the engineered yeast strain is a useful tool to validate candidate genes.

**FIG 5:**
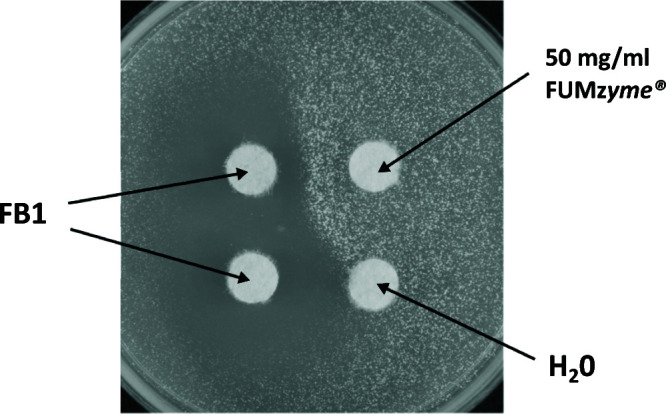
One hundred microliters of YTKT33 (OD_600_ = 0.3) was embedded in SC top agar after equilibration at 48°C and left at room temperature to solidify on SC media (bottom agar). Five microliters of crude FB1 (3,180 mg/L) was added to paper discs (left), while water or FUM*zyme* was added on the right.

The strain YTKT33 was also used in other applications, for instance, in the classical agar diffusion assay. When seeded into the top agar, application of a crude fungal extract containing FB1 applied to the plate (on a paper disc) produced a clear inhibition zone. As shown in Fig. 5, application of a detoxification enzyme (the commercial product FUM*zyme*) (37) next to the toxin-containing disc resulted in a diminished and deformed halo as the enzyme diffused into the inhibition zone and inactivated the toxin.

## DISCUSSION

Wild-type yeast is highly resistant to fumonisins, but we were successfully able to engineer a susceptible strain, YTKT33, by deleting several genes. Fumonisins are highly charged and water-soluble molecules (two tricarballylic acid side groups and a basic amine residue). An interesting, still unsolved question is how fumonisin enters the cell, as it is too polar to pass directly through lipid membranes. Obviously, yeast utilizes ABC transporters to reduce the concentration at the target through active afflux. We combined the described *yor1* mutation with a disruption of *SNQ2* (implicated in resistance to a broad range of mostly cationic compounds). In addition, we inactivated the ABC transporter encoded by *PDR12*, which is required for resistance to various weak acids. We settled for the triple mutant, yet these alterations may also sensitize the strain for other toxins or potentially so-far undescribed fumonisin synergists that might be present in crude extracts.

Fumonisin FB1 accounted for about 75% of the total B-type fumonisins in the crude extract. The additionally present FB2, FB3, and FB4 cannot fully explain the observed higher toxicity of the crude extract. In principle, other toxins or yet unknown low-toxicity substances acting as synergists of fumonisin might explain the higher toxicity of the crude extract. This effect limits the utility of semi-quantitative determination using the indicator strain in a bioassay. Since the inhibitory activity of the crude extract could be abolished by preincubation with FUM*zyme* (data not shown), the increased IC_50_ in the crude extract compared to pure fumonisin is likely due to substances that do not confer toxicity on their own but rather act as synergists of fumonisin.

Based on previous studies using mammalian cells, there does not appear to be any evidence for a synergism between fumonisin B_1_ and frequently co-produced *Fusarium* toxins, e.g., between the cyclic depsipeptides enniatins or beauvericin (38, 39). Potentially, moniliformin (40) or fusaproliferin might also be involved, which should be investigated in future work.

Medium composition and growth conditions have a strong influence on fumonisin toxicity. The binding of fumonisins, acting as competitive inhibitors of ceramide synthase, could be outcompeted by the concentration of the natural substrates of the enzyme that may be abundant, especially in rich media containing yeast extract. Particularly important in rich mediums was the targeting of the recycling pathway. As of yet, little is known about the degradation of complex sphingolipids and their reintegration into the biosynthesis pathway. Yeast produces inositol phosphosphingolipids, which can be hydrolyzed by the *ISC1* gene product (inositol phosphosphingolipid phospholipase C) back to phytoceramide and dihydroceramide, the products of ceramide synthase. This gene might be a candidate for an additional knockout, aiming to further decrease fumonisin resistance on YPD medium.

Yeast can be screened at high density on microtiter plates, where oxygen supply is low compared to growth on an agar surface. However, the slower (fermentative) growth under oxygen limitation seems to increase FB1 resistance. The concentration to inhibit growth was about one order of magnitude higher in SC liquid medium than in SC-URA agar plates.

The previously described fumonisin detoxification genes and a gene encoding an insensitive ceramide synthase all conferred FB1 resistance when expressed in YTKT33, validating the utility of the developed strain. The observed resistance level cannot be taken as direct evidence that one enzyme detoxifies better than the other. In a study where *Lemna minor* (duckweed) was exposed to HFB1 and deaminated FB1 (FPy1), the latter exhibited less toxicity than HFB1 (21). However, the final resistance level is not only caused by the reduced toxicity of the product but is also dependent on the catalytic properties and the expression level of the enzyme, which we have not determined in this study. Nevertheless, it is clear from our results that cytosolic *Sphingopyxis fumD* lacking the bacterial secretion signal can counteract a high level of FB1 entering the cell. It has been reported that hydrolyzed FB1 can be acylated and used as a substrate by ceramide synthases present in rat liver microsomes, forming toxic compounds (41). Under our experimental settings, even if N-acylation of HFB1 took place, it was insufficient to show toxicity. Hydrolysis of FB1 was also clearly shown to be a useful detoxification strategy in piglets through reduction of intestinal inflammation (37, 42).

Strain YTKT33 can be employed in the classical agar diffusion assay. Compounds that inhibit fumonisin biosynthesis at concentrations that are not inhibitory for growth have been described, e.g., pyrrocidine (43). The developed strain could be useful in screens for such substances or to study the mode of action of natural products or plant extracts that seemingly counteract fumonisin toxicity in ill-understood ways, e.g., reference (44). We have shown that by placing the commercially available detoxification enzyme FUM*zyme* next to the toxin, the deformed halo reveals the detoxification ability of the enzyme. In a more simple setup, preincubating amounts of FB1 with a detoxication agent and observing a diminished inhibition zone would be a possible embodiment of the utilization of the developed strain. Overall, the generated strain is clearly an improvement over previously reported indicator organisms (brine shrimp, hydra, duckweed), with the advantage that it can be used for heterologous expression of foreign genes and for high-throughput screening. It should be useful to identify currently unknown detoxification genes. For instance, it is unclear which gene encodes the fungal acetyltransferase converting FB1 to non-toxic acetylated fumonisin A_1_. Furthermore, there is a lack of knowledge about whether plant enzymes are involved in the production of hidden and bound fumonisins (45). The yeast indicator strain could be useful to rapidly test mutants of detoxification genes generated by site-directed mutagenesis or to select variants with superior kinetic properties from randomly mutagenized plasmids based on their ability to confer higher levels of resistance than the starting material. This can be achieved through high-throughput screening of the candidate genes using YTKT33. Potentially, novel genes could also be identified by expressing cDNA libraries and selecting resistance-conferring clones, similar to the approach leading to the identification of a deoxynivalenol-inactivating gene (46).

## MATERIALS AND METHODS

### Inactivation of ABC transporters in yeast

All yeast strains constructed in this work are derived from the yeast laboratory strains YPH500 (Matα, *ade2-101ochre, his3*Δ*200, leu2*Δ*1, lys2-801amber, trp1*Δ*1, ura3-52*) and YPH499 (47). For yeast gene nomenclature and composition of synthetic complete medium, see reference (48). Strains were routinely grown on YPD (1% yeast extract, 2% peptone, 2% dextrose, 2% agarose) and YPG (1% yeast extract, 2% peptone, 3% glycerol, 2% agarose) to identify respiratory competent cells. Information on yeast genes can be found in the Saccharomyces Genome Database [https://www.yeastgenome.org/, see reference (49)]. The strains and their construction are listed in Table 1. Single, double, and triple mutants with inactivated ABC transporters Snq2p, Pdr12p, and Yor1p were generated. *YOR1* was disrupted using the pDK30 plasmid kindly provided by Prof. Scott Moye-Rowley (University of Iowa). A restriction digest with BamHI + SacII released a 6.8 kb *yor1::hisG-URA3-hisG* fragment used for transformation (50). This disruption cassette was also used to transform strains YYM5 (*snq2*) and YRU75 (*pdr12*), as well as the *snq2 pdr12* strain YRE108 (51), which were kindly provided by Prof. Karl Kuchler (Medical University Vienna). Selection on 5-fluoroorotic acid was used for the removal of the *URA3* marker. *YOR1* disruption was confirmed via PCR screening using internal primers yor1ver_fw and yor1ver_rv, as well as through spotting on YPGE (1% yeast extract, 1% peptone, 3% glycerol, 2% agarose, 3% ethanol) plates containing 0.1 to 0.3 µg/mL oligomycin. Subsequent gene disruptions in the generated triple mutant strain YRU74 were performed using the Cre/*loxP* vector system (52). Plasmid pOS4a (galactose-inducible *GAL1* promoter-driven Cre recombinase and *ADE2* as selection markers) (53) was used to excise the *loxP*-flanked markers. Flanking and internal primers were used to screen yeast transformants (for primer sequences, see Table 2).

**TABLE 2:** Sequences of primers used in this study^*a*^

Primer name	Sequence (5′–3′)
yor1ver_fw	GGTTACGCTATTGGTGCATG
yor1ver_rv	GCAAGGAAAGTGACCAATG
dcka2-FW	ATGCCATTACCTCCGTCAACATTGAACCAGAAATCTAATAGAGTCTCGTACGCTGCAGGTCGAC
dcka2-RV	TTATTCAAACTTCGTTTTGAAAAACTTATGATCCATAGCCTCCTTCGCATAGGCCACTAGTGGATCT
cka2_scr-FW	CAAAGTTGGATATCCCTAATGACC
cka2_scr-RV	TTACTCAAAAGGTAAATGGCTCTCT
dlcb3-FW	ATGGTAGATGGACTGAATACCTCGAACATTAGGAAAAGAGCCAGGTCGTACGCTGCAGGTCGAC
dlcb3-RV	TTATGCTATATTTAAGAGGGAAAATAGGACGGGGCTGCACATTACCGCATAGGCCACTAGTGGATCT
lcb3_scr-FW	AAGCCTACGTTTTGGACTCTCA
lcb3_scr-RV	CATGGCCAGCACTATTTTCA
pUG72-REV	GCGTTTACCGTATCGCAGAATGG
pUG72-FWD	ATGGCCCAATCACAACCACATCTTAG
pUG73-REV	ATGCTATCGCCAAGGCTGTCAAGG
pUG73-FWD	GACACCTTCATCACCTAATTTCTCTTCAAC
VPS51_KO_ pUG_767_fw	GCGTATTTGCGGTGAGACGGAATCTGACGAGGATATTAAGTACAG cagctgaagcttcgtacgc
VPS51_KO_ pUG_767_rv	TCTCGAAGGAAGTGTTCGGTGAAAGCCACGATATGCCGCTGGAAA gcataggccactagtggatctg
KlLEU2 rev	GTTAGAAATGTCTTGGATGCAGGTG
KlLEU2fwd	CAGCAATGGCATTCAAGACCTTA
VPS51_KO_ upstream_ outside	ATAGGTGAGGCTCTGCATAG
VPS51_KO_ downstream_ outside	CGAAGGAAGTGTTCGGTGAAAGC
SmFumD_fw_ Leader	tcccaacgaccgaaaacctgtattttcagggatccATGAAAGAACACCAGTGTAG
SmFumD_fw_ noLeader_MAQ	tcccaacgaccgaaaacctgtattttcagggatccATGgctcaaactgacgacccaaa
SmFumD_rv	gtcttcaggagcgagttctggctggcttgcacgtgTTACTTGGATGGTTGACAAG
CYC1-RV	ggcgtgaatgtaagcgtgac
pVT-U_R4	GTGGCGAGAAAGGAAGGGAAGA
noHis_Seq_fw	caaaccggatcggactactagc

^*a*^ The underlined part of the sequences is complementary to the beginning or end of the resistance cassette amplified from different plasmids, while the non-underlined part is complementary to the 5′ or 3′ end of the gene to be knocked out.

Single deletions of candidate genes *CKA2* and *LCB3* were generated using plasmid pUG72 as a template to amplify the *loxP-KlURA3-loxP* cassette. Double mutants were obtained by using pUG73 as a template to generate a *loxP-KlLEU2-loxP* cassette. The resulting strain, YRU94 (*snq2*Δ*::hisG pdr12*Δ*::hisG yor1*Δ*::hisG cka2*Δ*::loxP-URA3-loxP, lcb3*Δ*::loxP-LEU2-loxP*), was used for further work. Marker loss was achieved via galactose induction after transformation with pOS4a. Subsequently, a red *ade2* mutant was picked from a YPD plate, indicating the loss of pOS4a in YRU94ML.

To inactivate *VPS51* in YRU94ML, primers VPS51_KO_pUG_767_fw and VPS51_KO_pUG_767_rv, providing the sequence overhangs for homologous recombination, were used to amplify a *loxP− Kluyveromyces lactis LEU2-loxP* construct from pUG73. The correct insertion of the marker (*vps51Δ::loxPKlLEU2-loxP*) in the transformants was confirmed via colony PCR using oligonucleotides flanking the disruption cassette (VPS51_KO_upstream_outside and VPS51_KO_downstream_outside) and primers contained within the *KlLEU2* gene (KlLEU2fwd and KlLEU2 rev), leading to strain YTKT1. Plasmid pBS49 (*GAL1-Cre URA3*) (54) was used for marker removal. The resulting strain after plasmid loss was designated YTKT33.

### Treatment with crude or purified fumonisins

The crude fumonisin extract used during the construction of strains was produced as follows: autoclaved maize media (10 g cracked maize, 1.9 g saccharose, 0.125 g casein hydrolysate, and 5 mL of water) in a 220 mL CultureJar G9 Glass Plant Tissue Culture Vessel (PhytoTech Labs) with Magenta B-Cap vented lids (Bioworld 309300051) were inoculated with conidia of various *Fusarium verticillioides* and *F. proliferatum* strains from our collections and incubated without shaking in the dark at 25°C for 29 days. After the addition of 25 mL of water, the cultures were homogenized, 25 mL of methanol was added, and extraction was performed by shaking for 30 minutes at 140 rpm at room temperature. Then, 1 mL aliquots were cleared by centrifugation (14,680 × *g* for 10 minutes at room temperature). Samples were diluted 1:400 with methanol/water (1:1) for analysis using high pressure liquid chromatography (HPLC). The remaining homogenates were frozen at −80°C until workup.

All homogenates containing ≥250 mg/L of FB1 were thawed, pooled, and centrifuged. Methanol (and partly water) was evaporated using a rotavapor at 35°C until precipitation occurred in the flask. The contents of the flask were centrifuged, and the precipitate was discarded. The fumonisin concentration of the remaining 160 mL of the red-brown, slightly viscous aqueous raw extract was determined, as described below, to be 5.3 g FB1/L. The extract was then slightly diluted and filter sterilized, and the working stock used in experiments was determined to contain 3.18 g/L FB1.

For the determination of fumonisins, an Agilent 1100 series HPLC was coupled to a Sciex 3500 triple quadrupole mass spectrometer. Separation was achieved on a Phenomenex Gemini C18 column (150× 4.6 mm, 5 µm particle size) at 30°C. Mobile phase A consisted of methanol/water/acetic acid (40/59.9/0.1, vol/vol/vol), while mobile phase B consisted of 0.1% acetic acid in methanol. The initial gradient (0% B) was maintained for 0.5 min before 73% B was reached at 5.9 min. The column was washed with 100% B from 6.0 to 9.9 min before re-equilibration with 0% B from 10.0 to 13.0 min. Then, 1 µL of samples (diluted with 50% aqueous methanol if needed) was injected into a flow of 800 µL/min. Ionization was achieved using a TurboV electrospray source in positive ion mode, applying 4,000 V of ionspray voltage at 550°C, curtain gas of 35 psi, and gas 1 and gas 2 settings of 50 psi each. Selected ion monitoring was employed with 20 ms dwell times for each transition, followed by 5 ms pause times. The following transitions were used [analyte: Q1 mass (Da) >Q3 mass (Da), declustering potential (V), collision energy (eV)]: FB1: 722.3 > 334.2 Da (91 V, 57 eV); FB1 qual: 722.3 > 352.2 Da (91 V, 50 eV); FB2/FB3: 706.3 > 318.3 Da (91 V, 55 eV); FB2/FB3 qual: 706.3 > 336.1 Da (91 V, 50 eV).

Solid FB1 was either provided by Dr. G. Jaunecker (Romer Labs, Tulln, Austria) or obtained from Fermentek (Jerusalem, Israel). For the comparison of the responses to different B-type fumonisins (Fig. 3), 1 mg of each of FB1, FB2, FB3 (>99%), and FB4 (>98%) was purchased from Fumizol Ltd. (Szeged, Hungary).

### Growth assays on solid and liquid media

Both complex (YPD) and SC (synthetic complete) media (47) supplemented with FB1 were used to conduct growth inhibition tests. For plate assays, liquid overnight cultures were diluted back to an OD_600 nm_ of 0.1. After reaching again about 0.3, they were diluted to an OD_600 nm_ of 0.1, 0.01, and 0.001, and 3 µL of these suspensions was spotted on the agar plates. Photographs were taken after incubation at 30°C for 5 days. For comparing the sensitivity to FB1 of different strains in liquid media, CELLSTAROneWell flat bottom plates (Greiner Bio-One GmbH, Kremsmünster, Austria) were placed in a Biotek Synergy H1 Hybrid microplate reader. Plates were incubated at 30°C for 24 h, and OD_600 nm_ was measured every hour using a fully automated set-up. A 0.6^N^ dilution series of the toxin was prepared manually by adding 120 µL of the previous toxin solution (200 µL in well 1) to 80 µL of prepared toxin-free media (in wells 2) and repeating this step in the following wells (wells 3–11) step by step. Then, 80 µL of inoculum was added to each well (1:1 dilution), leading to 11 different concentrations of FB1 and a no-toxin control (well 12). The initial OD_600_ of the strains was ~0.05 when diluted 1:1 with the FB1-YPD/SC mixture inside the well plate. Three replicates were used for each strain, and the growth after 24 h was analyzed by calculating the average of the OD_600_ of these replicates along with the standard deviation. The value of a blank (medium without yeast) was subtracted. In order to determine the response of the YTKT33 strain to different B-type fumonisins again, a dilution series of 0.6^N^ starting at a concentration of 500 µM was prepared in a microtiter plate and SC medium with 250 µM set as the highest one and wells containing only the yeast strain serving as a control. Four replicates were used, and the mean was calculated based on the OD values measured after 24 h, along with the standard deviation.

### Expression of candidate genes

Plasmid pYes2-P_TEF1_ was used to express different candidate genes expected to confer resistance against FB1. It contains *URA3* as a selection marker, the *TEF1* promoter, and a N-terminal 6xHis-tag (33). The empty vector, as well as one containing the *Fusarium verticillioides* self-resistance gene (33), *FUM18,* as a positive control, were kindly provided by Dr. Vito Valiante (Leibniz Institute for Natural Product Research and Infection Biology, Hans Knöll Institute, Jena, Germany). The reading frames of other genes of interest were PCR-amplified using a polymerase with proof-reading activity (Phusion High-Fidelity DNA Polymerase, Thermo Fischer Scientific, Vienna, Austria) and about 50-bp-long primers with 35-bp homology to the plasmid and the gene at both the 5′ and 3′ ends. The candidate genes were recombined behind the *TEF1* promoter, and the resulting plasmids were recovered from yeast and transformed into *Escherichia coli* by electroporation. For sequence confirmation, primers pVT-U_R4 (see Table 2) and CYC1-RV were used.

The original *Sphingopyxis sp.* MTA144 *fumD* esterase gene recoded for *Pichia* expression (34) was used as a template for PCR amplification. For generating the construct with the natural bacterial secretion signal, primers SmFumD_rv and SmFumD_fw_Leader were combined. In order to generate the *fumD* construct without a leader, primers SmFumD_rv and SmFumD_fw_noLeader_MAQ were used. The respective PCR products were co-transformed with the BamHI + Eco721 cut vectors for *in vivo* recombination. The *Aspergillus* amine oxidase gene as described (35) was ordered from BioCat (Heidelberg, Germany), flanked by a sequence containing BamHI and Eco721 for cloning into vector pYes2-P_TEF1_, yielding pTAK37. Modifications were added to this plasmid later on to test for potential negative effects of the 6xHis-tag. Therefore, pTAK37 was cut with BamHI and PvuII (removing the 6xHis-tag) and blunt ends religated after Klenow-fill in. This vector, pTAK38, was used to clone other detoxification genes and transformed into YTKT33 for spotting.

### Agar diffusion test

Then, 6 mL of SC top agar equilibrated at 45°C was combined with 100 µL of YTKT33 (OD_600_ = 0.3) and poured into 60 mm-diameter petri dishes containing SC bottom agar. Sterile antibiotic discs containing either FB1 (5 µL of “crude FB1” containing 3.18 mg/L FB1), water (control), or FUM*zyme* from Biomin (5 µL of 50 mg/mL) were placed on the solidified dry surface.

## Data Availability

The sequences of the recoded Sphingopyxis fumD and Aspergillus amine oxidase genes are given in the supplementary material file. fumD accession: D2D3B6, Aspergillus amine oxidase accesion: A2R252. Strain requests (by means of a material transfer agreement) should be directed to FFoQSI (juergen.marchart@ffoqsi.at).

## SUPPLEMENTAL MATERIAL

Recoded genes aem.01211-23-s0001.docx Gene sequences.

ASM does not own the copyrights to Supplemental Material that may be linked to, or accessed through, an article. The authors have granted ASM a non-exclusive, world-wide license to publish the Supplemental Material files. Please contact the corresponding author directly for reuse.
