# Supplementary material for: Development of a fumonisin-sensitive Saccharomyces cerevisiae indicator strain and utilization for activity testing of candidate detoxification genes
Source: Appl Environ Microbiol. 2023 Dec 6;89(12):e01211-23. doi: 10.1128/aem.01211-23 (PMC10746191; doi:10.1128/aem.01211-23)
Supplement: Recoded genes — Gene sequences. [file aem.01211-23-s0001.docx]

LOCUS fumD_GeneArt_codon-optimised_Pichia 1656 bp DNA linear

1 GGTACCCTCG AGAAAAGAAT GAAAGAACAC CAGTGTAGAG GTGGTAGAGC TTCTCCAGCT
 61 GCTCCAGCTA CTTGGCTTGC TAGAATCTCC GTTTCCCGTG GTGCTTCTGC TATTGCTTGG
 121 ACTTTCATGT TGGGTGCTAC TGCTATTCCA GTTGCTGCTC AAACTGACGA CCCAAAGCTT
 181 GTTAGACACA CTCAATCTGG TGCTGTTGAA GGTGTTGAGG GTGACGTTGA GACTTTCTTG
 241 GGTATCCCAT TTGCTGCTCC ACCAGTTGGT GATTTGAGAT GGAGACCACC TGCTCCACCA
 301 AGAGCTTGGG CTGGTACTAG AGATGGTAGA AGATTCGCTC CAGACTGTAT CGGTAACGAG
 361 AGATTGAGAG AGGGTTCAAG AGCTGCTGGT ACTTCTGAGG ACTGTTTGTA CTTGAACATC
 421 TGGTCCCCAA AGCAAGTTGG AAAGGGAGGA TTGCCAGTTA TGATCTGGGT TTACGGTGGT
 481 GGTTTTTCTG GTGGTTCCGG TGCTGTTCCA TACTACGACG GTTCTGCTTT GGCTCAAAAG
 541 GGTGTTGTTG TTGTTACTTT CAACTACAGA GCTGGTATCT TGGGATTTTT GGCTCACCCA
 601 GCTTTGTCTA AAGAATCCCC AAACGGTGTT TCTGGTAACT ACGGTTTGTT GGACATGTTG
 661 GCTGCTTTCA AGTGGGTTCA GAACAACATC AGAGAATTTG GTGGTGACCC AAACAGAGTT
 721 ACTGTTTTCG GTGAATCTGC TGGTGCTTCT GCTTTGGGTT TGTTGTTGAC TTCTCCATTG
 781 TCTGAGTCCG CTTTCAACCA GGCTATTTTG CAATCTCCAG GATTGGCTAG ACCATTGGCT
 841 ACTTTGTCTG AATCTGAGGC TAACGGATTG GAATTGGGTG CTGACATTTC CGCTTTGAGA
 901 AGAGCTGATG CTGGTGAGTT GACTAAGATC GCTCAGTCCA GAATCCCAAT GTCCAGACAG
 961 TTCACTAAGC CAAGACCAAT GGGTCCAATC TTGGACGGTT ACGTTTTGAG AACTTTGGAC
1021 GTTGACGCTT TTGCTAAGGG AGCTTTCAGA AAGATCCCAG TTTTGGTTGG TGGTAACGCT
1081 GATGAAGGTA GAGCTTTCAC TGACAGATTG CCTGTTAAGA CTGTTTTGGA GTACAGAGCT
1141 TACTTGACTG AGCAATTCGG TGATGAAGCT GACGCTTGGG AAAGATGTTA CCCAGCTAAC
1201 TCTGATGCTG ATGTTCCAGC TGCTGTTGCT AGATTGTTCG GTGACTCCCA GTTCAACAAC
1261 GGTATCGAGT TGTTGTCTGC TGCTTTCGCT AAGTGGAGAA CTCCATTGTG GAGATACAGA
1321 TTCACTGGTA TCCCAGGTGC TGGTAGAAGA CCAGCTACTC ACGGTGACGA GATCCCATAC
1381 GTTTTCGCTA ACTTGGGACC ATCTTCCGTT TCTATGTTCG GTTCTTTGGA AGGTGGAGCT
1441 GGTGCTTCCG ATATCAAGTT GGCTACTGAG ATGTCAGCTG CTTGGGTTTC CTTTGCTGTT
1501 CACGGTGTTC CAGACCAAGG TACTAAGTCT CACTGGCCAA GATTCGAGAG AAGAGGTGAA
1561 ATCATGACTT TCGGTTCCCA AGTTGGTTCT GGTGAAGGAT TGGGAGTTTC TCCATCCAAG
1621 GCTTGTCAAC CATCCAAGTA ATAATCTAGA GAGCTC
//

ACCESSION D2D3B6

MKEHQCRGGRASPAAPATWLARISVSRGASAIAWTFMLGATAIPVAAQTDDPKLVRHTQSGAVEGVEGDVETFLGIPFAAPPVGDLRWRPPAPPRAWAGTRDGRRFAPDCIGNERLREGSRAAGTSEDCLYLNIWSPKQVGKGGLPVMIWVYGGGFSGGSGAVPYYDGSALAQKGVVVVTFNYRAGILGFLAHPALSKESPNGVSGNYGLLDMLAAFKWVQNNIREFGGDPNRVTVFGESAGASALGLLLTSPLSESAFNQAILQSPGLARPLATLSESEANGLELGADISALRRADAGELTKIAQSRIPMSRQFTKPRPMGPILDGYVLRTLDVDAFAKGAFRKIPVLVGGNADEGRAFTDRLPVKTVLEYRAYLTEQFGDEADAWERCYPANSDADVPAAVARLFGDSQFNNGIELLSAAFAKWRTPLWRYRFTGIPGAGRRPATHGDEIPYVFANLGPSSVSMFGSLEGGAGASDIKLATEMSAAWVSFAVHGVPDQGTKSHWPRFERRGEIMTFGSQVGSGEGLGVSPSKACQPSK

The leader sequence is highlighted in yellow

Codon optimized (for yeast expression) *Aspergillus* amine oxidase gene (ACCESSION A2R252):

ATGTCTGTTTCTAACGATCCAACTACTAAATTGTACGATGCTGTTATTGTTGGTGCTGGTTTGTCTGGTTTGCAAGCTGCTCATTCTATTCAAGCTGCTGGTTTTTCTGTTTGTATTTTGGAAGCTACTGATAGAATGGGTGGTAAAACTTTGACTGTTAAGTCTTCTGAAAAGGGTTACAACGATTTGGGTGCTGCTTGGGTTAATGATACTAACCAAACTGAAATTTTCAAGTTGCATCAAAGATACGGTTTGGATGGTGTTGTTCAATACACTTGTGGTGACGATATTTTGGAATCTGGTGAAGGTGTTATTAGAAAGATTCCTTATGGTTTGCCATTGACTGGTTTGCCAAAGAAGTTGTTGGATATTTTGAGAATTGAGTCTTCTAGATTGGATTTGGATGATCCAACTTCTTTCCCTGGTGCTACTGAAGTTGATAATTTGACTTTTAGAGACTTCTGTGTTGAAAAGACTGGTTCTGAAGATGTTATTCATATTACTGATGCTATCTCTACTGCTTTGTTGGGTTTGAACTCTAATGAATTGTCTGCTTTGTATATGTTGTACTACTTCAAGTCTGGTTCTGGTATTGATAACTTGTTGTCTGATGAAAGAGATGGTGCTCAATACTTGAGAACTAGACAAGGTACTCAAACTATTGCTAGAAAAATGGCTGATGAATTGACTCAATCTGATATTTTTCTGGGTATGCCTGTTACTTCTATTAACCAAACTGATGCTGATGCTCATTGTGTTGTTCAAACTTTGGATGGTTCTTCTTTCAGATGTAGAAGAGTTATTGTTTCTATCCCTACTACTTTGTACAGATCTGTTTCTTTCCATCCTCCTTTGCCTCATGCTAAGCAAGTTTTGTCTGATCATACTATTATGGGTTATTACTCTAAGGTTATCTTCATTTTCAAGGAACCTTGGTGGAGAGATGCTGGTTTGACTGGTATTGTTAACTGTGCTGGTGGTCCTATTACTTTTACTAGAGATACTTCTGTTCCAACTGATGATCAATGGTCTATTACTTGTTTTATGGTTGGTTCTAGAGGTAGAGCTTGGTCTAAATTGTCTAAGGATGATAGATACTCTCAAGTTTGGGAACAATTCAGAAGATGTTTTGAAGAATTTGTCGAAAACATCCCAGAACCTGTTAACACTTTGGAAATGGAATGGTCTAAGGAACCTTACTTTTTGGGTGCTCCTTGTCCAGCTATGATTCCAGGTTTGTTGACTACTGCTGGTTCTGATTTGGCTGCTCCACATGGTAAAGTTCATTTTATTGGTACTGAAACTTCCACTGTTTGGAGAGGTTATATGGAAGGTGCTATTAGAGCTGGTCAAAGAGGTGGTGCTGAAGTTGTTACTGCTTTGCAAGAAGATTAA
